# Two-Step Single Slope/SAR ADC with Error Correction for CMOS Image Sensor

**DOI:** 10.1155/2014/861278

**Published:** 2014-01-22

**Authors:** Fang Tang, Amine Bermak, Abbes Amira, Mohieddine Amor Benammar, Debiao He, Xiaojin Zhao

**Affiliations:** ^1^College of Communication Engineering, Chongqing University (CQU), China; ^2^Hong Kong University of Science and Technology (HKUST), ECE Department, Clear Water Bay, Kowloon, Hong Kong; ^3^School of Computing, University of the West of Scotland, UK; ^4^Qatar University, Qatar; ^6^School of Mathematics and Statistics, Wuhan University, Wuhan, China; ^5^College of Electronic Science and Technology, Shenzhen University, Shenzhen, China

## Abstract

Conventional two-step ADC for CMOS image sensor requires full resolution noise performance in the first stage single slope ADC, leading to high power consumption and large chip area. This paper presents an 11-bit two-step single slope/successive approximation register (SAR) ADC scheme for CMOS image sensor applications. The first stage single slope ADC generates a 3-bit data and 1 redundant bit. The redundant bit is combined with the following 8-bit SAR ADC output code using a proposed error correction algorithm. Instead of requiring full resolution noise performance, the first stage single slope circuit of the proposed ADC can tolerate up to 3.125% quantization noise. With the proposed error correction mechanism, the power consumption and chip area of the single slope ADC are significantly reduced. The prototype ADC is fabricated using 0.18 **μ**m CMOS technology. The chip area of the proposed ADC is 7 **μ**m × 500 **μ**m. The measurement results show that the energy efficiency figure-of-merit (FOM) of the proposed ADC core is only 125 pJ/sample under 1.4 V power supply and the chip area efficiency is 84 k **μ**m^2^ *·*cycles/sample.

## 1. Introduction

Column-parallel readout scheme is wildly applied in high resolution CMOS image sensor designs. Compared with arraywise ADC schemes, column-parallel readout circuit could be realized with mediate speed, low power ADC, whose channel has much simple design complexity. Column-parallel single slope ADC is the most popular scheme used in the mass produced consumer electronic devices [1]. Although the architecture of the single slope ADC is very simple, the operation speed is low as well as the energy efficiency; thus, it is difficult to be applied in a high speed CMOS image sensor. SAR ADC is one of the best energy efficient ADC schemes [2]. However, in order to guarantee the linearity, SAR ADC either requires large chip area or calibration [3]. Although split capacitor technique can significantly reduce the total capacitance, capacitor array is still difficult to be matched for a quantization resolution larger than 10 bits. Cyclic ADC has similar operation speed as the SAR ADC which can generate *N*-bit output code with *N* quantization cycles [4]. The drawback of the cyclic ADC is that its performance is highly dependent on the amplifier design, which consumes large power. Two-step single slope ADC was proposed in the prior art [5, 6] in order to solve the operation speed issue of the traditional single slope ADC. However, multiramp signals are required for the proposed scheme and the first single slope ADC must meet a full resolution noise specification.

In this paper, we report an 11-bit hybrid ADC for CMOS image sensor. The quantization circuit consists of a single slope ADC and a SAR ADC. The single slope ADC generates 3-bit quantization code and 1 redundant bit. After combining with the following 8-bit SAR ADC code using the error correction algorithm, the proposed 11-bit ADC could tolerate up to 3.125% of single slope quantization noise. The power consumption and chip area of the single slope ADC can be significantly reduced.

This paper is organized as follows.  Section 2 introduces the related prior art.  Section 3 describes the proposed ADC with error correction mechanism and the ADC circuit implementation.  Section 4 demonstrates the prototype experimental results.  Section 5 delivers the conclusion.

## 2. Related Work

**Figure 1:**
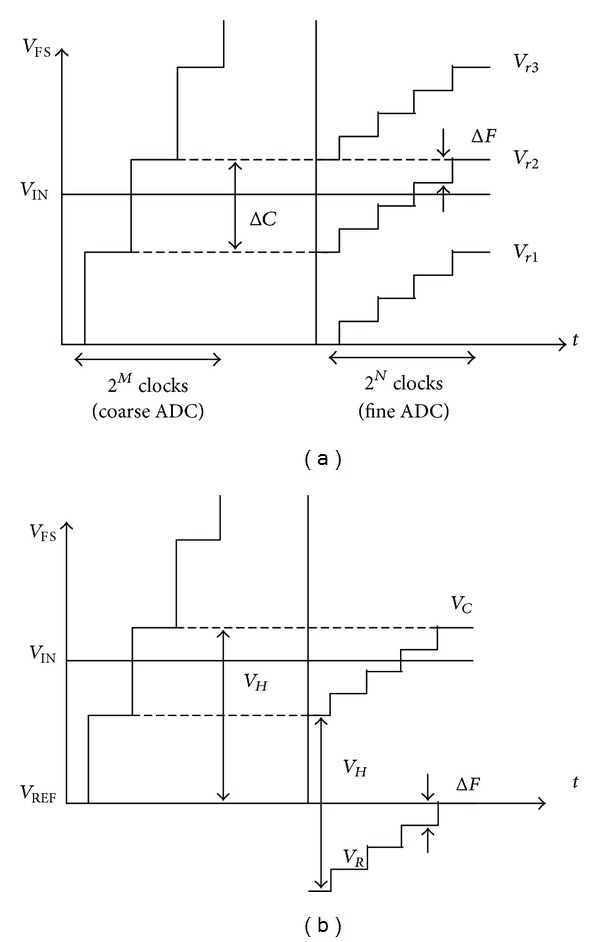
The principle of the conventional two-step single slope ADC [7].

The principle of the conventional two-step single slope ADC is shown in Figure 1 [7]. Firstly, the input signal is quantized by the 3-bit single slope ADC. Then, the quantized 3-MSB code selects a certain reference from 8 slope voltages, if assuming that *M* = 3 and *N* = 8. Secondly, the input signal is quantized by the following 8-bit single slope ADC and the final output code is generated by combing the 11-bit code.

**Figure 2:**
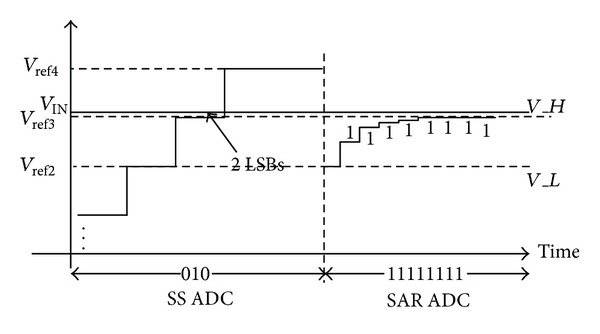
The example of how missing code is generated by the first stage ADC noise.

Although the two-step single slope ADC increases the quantization speed by a factor of 10 compared with the traditional scheme, about 100 quantization cycles are still required. In order to further increase the energy efficiency, an 8-bit SAR ADC could be adopted in the second ADC stage instead of another 8-bit single slope ADC. Thus, the number of quantization cycles can be reduced from 100 to less than 25. Although two-step ADC can increase the quantization energy efficiency, either two-step single slope or single slope/SAR scheme has a drawback that the first single slope ADC requires full resolution noise performance.  Figure 2 shows an example, where the input signal is set to 2-LSB higher than *V*_ref3_.

If we assume that the quantization noise of the first 3-bit single slope ADC is larger than 2-LSB, the output quantization result of the first ADC stage probably equals “010” instead of the ideal value “011.” Then, *V*_ref2_ and *V*_ref3_ are selected as the high reference voltage *V*_*H*_ and low reference voltage *V*_*L*_, respectively, for the following SAR ADC quantization. Since the input signal *V*_IN_ is 2-LSB higher than *V*_*H*_, the SAR ADC enters into the saturation region and finally an upper limit code “11111111” is generated. After combing these two codes, a quantization result “01011111111” is generated which is 2-LSB smaller than the expected output “0110000010.” As a result, missing code happens even if the SAR ADC is perfectly implemented.

## 3. Proposed ADC with Error Correction Mechanism

Designing an ultralow noise single slope ADC could solve the missing code problem; however, perfect single slope ADC requires large power consumption and chip area for the comparator design. Another way to solve the missing code issue is introducing assistant algorithm such as digital error correction mechanism.

**Figure 3:**
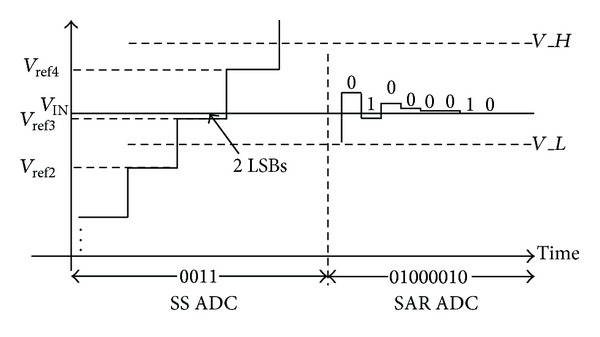
The operating principal of the proposed ADC without quantization noise.

The proposed two-step single slope/SAR ADC principal is shown in Figure 3. The single slope ADC generates 4-bit code which consists of 3-MSB code and 1 redundant bit. The 4-bit code selects reference voltages *V*_*H*_ and *V*_*L*_ for the SAR ADC, where *V*_*H*_ − *V*_*L*_ = *V*_ref_*n*__ − *V*_ref_*n*−2__. Assuming that the first stage single slope quantization noise could be ignored, the 4-bit output code should be “0011.” After SAR quantization, a “01000010” code is generated. Then, the redundant bit “1” is recombined with the SAR ADC code by using the error correction algorithm as shown in (1). Finally, the proposed ADC generates an ideal digital result “00111000010” (450):

(1) 0010+11000010=00111000010.

**Figure 4:**
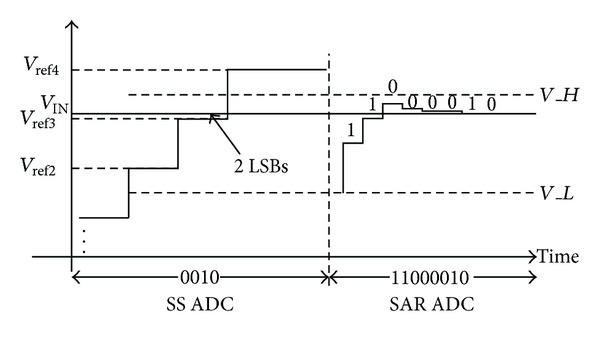
The operating principal of the proposed ADC with quantization noise.

If the quantization noise of the first stage single slope ADC cannot be ignored, the 4-bit output code is “0010,” as shown in Figure 4. The 4-MSB code selects other *V*_*H*_ and *V*_*L*_, which are one-step voltage lower than the case shown in Figure 3. After SAR quantization, “11000010” is generated. Similarly, the 3-MSB, the 1 redundant bit “0010,” and “11000010” are recombined as “00111000010” (450) with the error correction algorithm being as shown in (2). It is clear that the final results both with and without quantization noise are the same. Thus the missing code due to the first stage ADC quantization noise is eliminated by adopting the error correction mechanism. The proposed error correction algorithm can be expressed as in (3):

(2) 0011+01000010=00111000010,

(3) $$\begin{array}{c} D_{\text{out}}=D\left[11:8\right]2^{7}+D\left[7:0\right]. \end{array}$$

**Figure 5:**
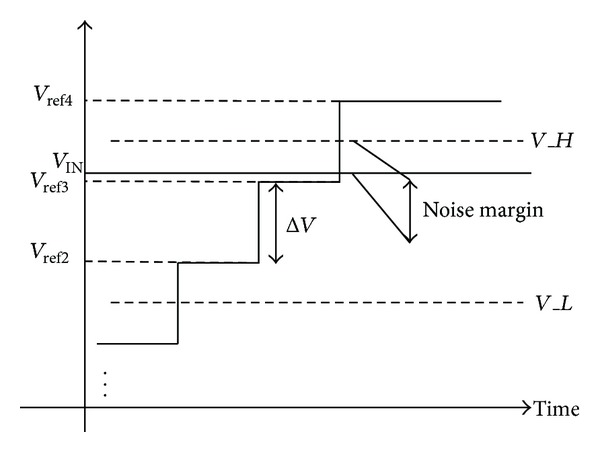
The noise margin definition of the proposed error correction mechanism.

The noise margin is defined as the voltage difference between the input voltage *V*_IN_ and *V*_*H*_, where Δ*V* equals *V*_*pp*_/2^4^. The maximum noise margin is Δ*V*/2, when the single slope ADC quantization noise can be ignored. Therefore, the error correction algorithm can tolerate up to Δ*V*/2 first stage quantization noise. If the input signal has 1.2 V voltage range *V*_*pp*_ up to 37.5 mV (3.125%) single slope ADC noise can be corrected (Figure 5).

**Figure 6:**
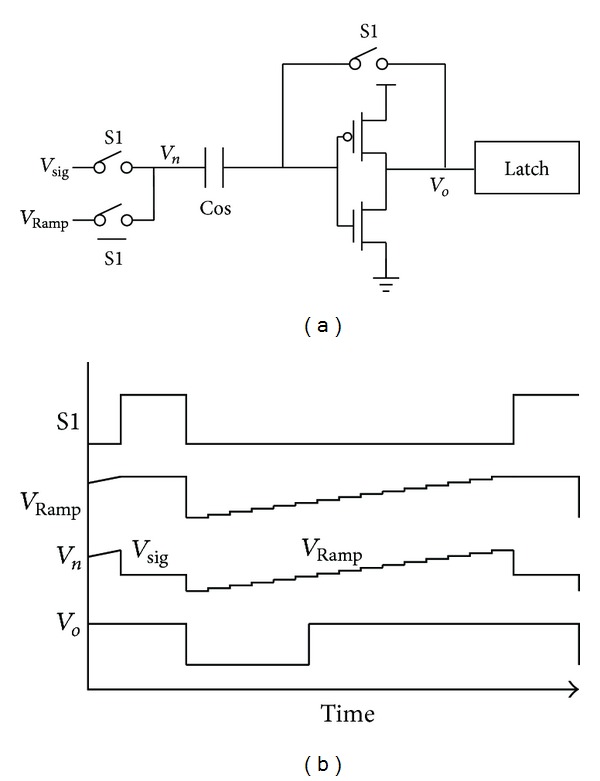
The schematic and operating timing sequence of the single slope ADC.

Since the single slope ADC has a large noise margin, the design complexity can be significantly relaxed. This work adopts an ultralow power single slope ADC scheme as shown in Figure 6. Two main input referred noise sources of the single slope ADC include the KT/C noise introduced by capacitor Cos during reset phase (S1) and the random noise of inverter transistors. A transient noise simulation is performed with *Cos*⁡ = 50 fF and 1.2 V power supply. The simulated noise frequency band is up to ×1000 clock frequency. Minimum transistor size is applied in order to reduce the short circuit current consumption. 50 simulations indicate that the peak input refereed single slope ADC noise is smaller than 18 m*V*_*pp*_ while maintaining more than 1/2 noise margin. Although the capacitor Cos can be further scaled down, the saved chip area is insignificant when the entire 11-bit ADC area is taken into account.

**Figure 7:**
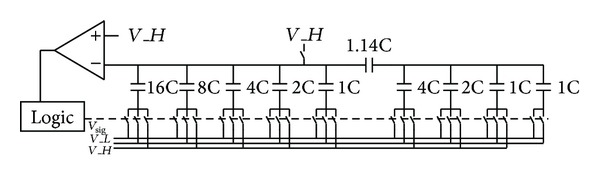
The schematic of the SAR ADC.

The SAR ADC takes charge of the 8-bit LSB quantization. With standard structure as described in [2], more than 128 unit capacitors are required, leading to an unaffordable chip area. In order to reduce the capacitor array size, this work adopts 5-bit/3-bit split capacitor scheme, where only 32 unit capacitors are required. The schematic of the SAR ADC is shown in Figure 7. According to the Monte-Carlo simulation, the SAR ADC can achieve 8-bit linearity with 30 fF and 5 *μ*m × 5 *μ*m metal-insulator-metal (MIM) unit capacitor.

## 4. The Experimental Result

**Figure 8:**
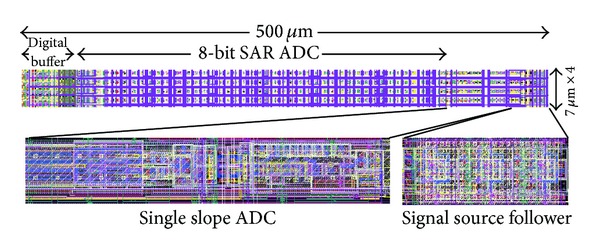
The fabricated chip layout of the proposed ADC.

The proposed two-step single slope/SAR ADC is fabricated using 0.18 *μ*m CMOS process and the chip layout is shown in Figure 8. Each ADC channel occupies 7 *μ*m × 500 *μ*m chip area in order to integrate more than 1000 channels in an ADC array for high pixel resolution CMOS image sensor applications. The signal source follower is used to buffer the pixel analog voltage. The digital buffer is designed to scan out the ADC array digital output data. Therefore, the ADC core only occupies less than 7 *μ*m × 400 *μ*m area and the first stage single slope ADC requires only 13% chip area of the ADC core.

**Figure 9:**
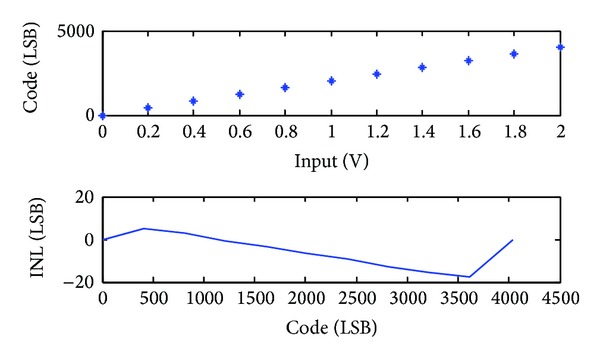
The measured INL of the single slope ADC.

**Figure 10:**
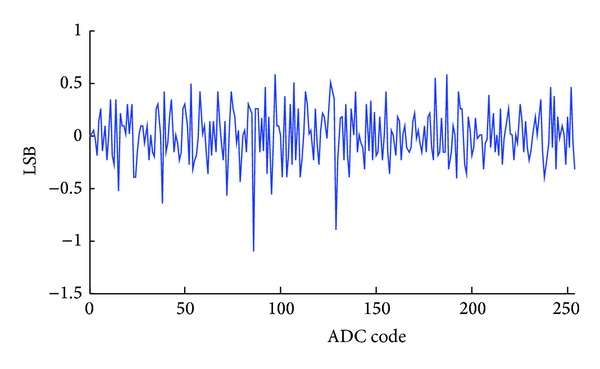
The measured DNL of the SAR ADC.

**Figure 11:**
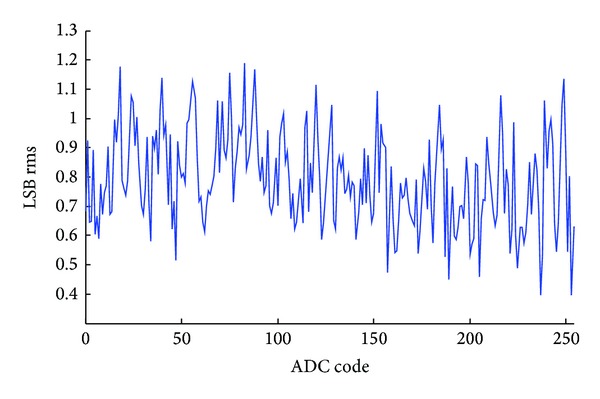
The measured SAR ADC output noise.

The integrated nonlinearity (INL) of the first stage single slope ADC is shown in Figure 9. The nonlinearity indicates the input offset voltage of the single slope ADC. According to Figure 9, about 20 LSBs systematic offset voltage exists for 12-bit quantization resolution. This offset voltage is much less than 37.5 mV, which can be easily corrected by the error correction algorithm without degrading the ADC performance. The differential nonlinearity (DNL) of the SAR ADC is shown in Figure 10. A +0.5/−1 LSB DNL is achieved leading to an 8-bit linearity. Only two DNL tones are −1 LSB, while others are located within +0.5/−0.5 LSB region. The SAR ADC output random noise is measured as shown in Figure 11. With 10,000 samples, the SAR ADC indicates a standard deviation random noise about 1.2-LSBrms. Since the DAC reference voltage is 150 mV for a 1.2 V input signal range, 1.2-LSB refers to 0.7 mVrms quantization noise.

**Figure 12:**
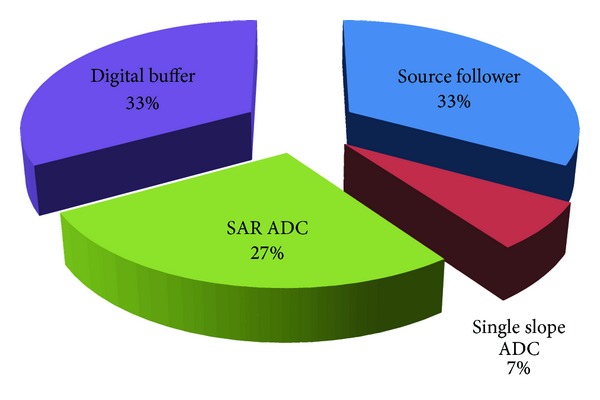
The measured power consumption distribution of the column-parallel circuit.

The power consumption distribution of the whole column-parallel circuit is shown in Figure 12, including the source follower, ADC, and scan buffer. The power consumption of the proposed two-step single slope/SAR ADC is 5 *μ*W with 1.2 V power supply under 40 Ksamples/s. The single slope ADC consumes 1 *μ*W, while the SAR ADC consumes the rest 4 *μ*W. The specification of the proposed ADC is summarized with some other prior arts in CMOS image sensor field as shown in Table 1. Two figure-of-merits (FPM) are compared including the energy efficiency and chip area efficiency. The energy efficiency of the proposed ADC could be estimated as 125 pJ/sample. The chip area efficiency is defined as the chip area × quantization cycles per sample, resulting in 84 k *μ*m^2^·cycles/sample of this work.

## 5. Conclusion

This paper presents a high energy efficiency high chip area efficiency column-parallel ADC for CMOS image sensor applications. The proposed ADC consists of a single slope ADC and a SAR ADC. The single slope ADC generates 3-MSB code and 1 redundant bit, which is combined with the following 8-bit SAR ADC quantization result using the proposed error correction algorithm. Up to 3.125% quantization noise of the first stage ADC can be tolerated; thus, the chip area and power consumption of the single slope ADC are significantly reduced. The ADC prototype is fabricated using 0.18 *μ*m CMOS process. Measurement results show a 125 pJ/sample core energy efficiency and 84 k *μ*m^2^·cycles/sample chip area efficiency.

## Figures and Tables

**Table 1:** The summarized comparison with other types of column-parallel architectures.

Specification	This work	Two-step SSADC [5]	Cyclic ADC [4]	Calibrated SAR ADC [3]
Process	0.18 µm	0.25 µm	0.25 µm	0.18 µm
Quantization resolution	11-bit	10-bit	12-bit	10-bit
Chip area	7 × 500 µm^2^	7.4 µm pitch	40 × 2200 µm^2^	14 × 700 µm^2^
Power consumption	5 µW at 40 Ksamples/s	75 µW at 60 Ksamples/s	1.9 mW (full chip) at 1.9 Msamples/s	1.5 mW (full chip) at 2 Msamples/s
Quantization cycles	24 cycles/sample	>160 cycles/sample	12 cycles/sample	13 cycles/sample
DNL	+0.5/−1 LSB	<+/−1 LSB	+0.76/−0.81 LSB	0.34 LSB
FOM1 Energy efficiency	125 pJ/sample ADC core	1250 pJ/sample ADC core	1 nJ/sample full chip	750 pJ/sample full chip
FOM2 Area efficiency	84 k µm^2^·cycles/sample	—	1056 k µm^2^·cycles/sample	127 k µm^2^·cycles/sample
